# New Developments in Physical Education and Sport

**DOI:** 10.3390/ijerph17249171

**Published:** 2020-12-08

**Authors:** Antonio Granero-Gallegos

**Affiliations:** Department of Education and Health Research Center, University of Almería, 04120 Almeria, Spain; agranero@ual.es

## 1. Introduction

Continuous updates of knowledge among professionals in physical education (PE) and sport are essential for the goal of developing quality professional work. In our current globalized and changing world, continuous and permanent learning is fundamental for organizing and complementing initial training and previous experience.

To ensure competence in the field of PE and sport, it is important to have a proactive attitude towards the extensive knowledge arising from continuous research and to integrate it with one’s prior knowledge and work experience. This is the path to career improvement and satisfaction. Certainly, research is an unfinished and diverse construct: it is a permanent learning process in terms of interpretations, explanations, and contributions.

Globalization, research, and education must respond to continuous changes in the different spheres of social, economic, and scientific activity. Information and communication technologies provide excellent mechanisms to facilitate the study, exchange, and dissemination of principal research findings regarding knowledge and knowledge socialization events. For this reason, and to develop competence or learning, it is also necessary to select the most appropriate information as well as quality publications that are produced with methodological rigor. Integrating knowledge in a way that can be suitably applied in the modern setting will help us to flourish as highly competent professionals in PE and sports, a field that is increasingly taken up by the population.

### The Current Special Issue

This Special Issue was proposed in order to compile some of the latest research advances in the PE field and to evaluate the relationship of different variables with physical activity behaviors outside the classroom. Improving teaching processes and understanding the different psychological variables that affect learning are of continuous concern among the different agents involved in teaching. This is highlighted by the wide range of articles in the educational context (primary, secondary, and higher education) that focus on such issues as innovative teaching methodologies, pedagogical models, motivation, satisfaction and frustration of basic psychological needs, perfectionism, self-esteem, motivational climate, and emotional intelligence.

This issue also includes research on different aspects for promoting moderate and vigorous physical activity in students, both inside and outside of the educational center, and for creating healthy and permanent physical exercise habits in the future. Similarly, research focused on activities in nature, recreational pursuits, and sports tourism have been published, with samples comprising students and healthy adults.

Numerous and varied articles on the practice of physical activity and sports by special populations are featured in this Special Issue. On the one hand, we explore studies carried out in different sports contexts, such as those conducted on football players, young professional athletes, handball referees, and professional endurance athletes. On the other hand, we include studies on special populations such as elderly people; these articles show the importance of leading an active life or are focused on the most avant-garde technological advances in physical activity, such as a pro-device for monitoring physical activity and movement.

The progress made in adapting measurement instruments is also assessed in this Special Issue, especially the implications of their use for future research; four articles that focus on different characteristics of instruments’ psychometric properties have been published. Furthermore, in recognition that physical and sports education of high quality must be offered to society and must increasingly be based on empirical scientific evidence, this Special Issue also includes articles that report systematic review and meta-analysis, as well as studies that use experimental and quasi-experimental methodologies.

The topics covered by the articles are diverse, as are the methodologies used, and we are pleased that new developments in PE and sport have aroused interest in the scientific community. Our aim is to contribute to advances of the scientific debate and to provide a quality update for different professionals in this field.

## 2. The Studies Included

We received a total of 42 submissions, of which 28 were ultimately accepted. The submission process was open from October 2019 to September 2020. As readers will see, most of the accepted publications used cross-sectional methodologies, although qualitative, experimental, quasi-experimental, and meta-analytical studies were also included, along with psychometric instrument validations and systematic reviews. The majority of the studies were conducted in Spain, although some studies were conducted in populations from Taiwan, Poland, Luxembourg, Germany, Turkey, Lithuania, Croatia, Mexico, and Portugal.

Presented in chronological order of publication, this Special Issue includes the papers described below.

Trigueros, Aguilar-Parra, López-Liria, and Rocamora [1] used structural equation modeling to analyze the influence of several psychological control variables on emotional intelligence in a large sample of 1602 secondary school students. They also examined the meta-cognitive strategies employed by students with regard to emotional intelligence and the thwarting of basic psychological needs. Their results showed that psychological control positively predicted each of the sub-factors related to the thwarting of psychological needs, whereas the thwarting of psychological needs negatively predicted emotional intelligence, and emotional intelligence positively predicted meta-cognitive thinking. As the authors note, this research supports the tenets of self-determination theory, viewed from the darker side, while introducing new variables and demonstrating their applicability to Spanish culture.

Granero-Gallegos, Ruiz-Montero, Baena-Extremera, and Martínez-Molina [2] used multi-level regression models to analyze the effects of perceived teaching competence, motivation, and basic psychological needs on disruptive behaviors in secondary school PE students. Their results revealed that disruptive behaviors were more likely to occur among boys and that misbehavior decreased when a teacher was perceived as competent. Furthermore, students with greater self-determined motivation were more likely to exhibit fewer behaviors related to low engagement and irresponsibility, whereas amotivation increased various disruptive behaviors in the classroom.

Fuentesal-García, Baena-Extremera, and Sáez-Padilla [3] carried out two different research studies to analyze the psychometric properties of the Physical Activity Enjoyment Scale applied to different contexts, for initial or original use, such as physical activity in nature. This included a confirmatory factorial analysis. The authors concluded that this scale could not be applied as-is in the studied context and that certain items had to be eliminated and/or modified. From this work, they obtained a new specific instrument for this type of practice.

Hinojo, López, Fuentes, Trujillo, and Pozo [4] carried out experimental research on flipped learning as an innovative approach to physical education teaching and learning processes. The authors evaluated the effectiveness of flipped learning compared with the traditional methodology. Two study groups were established: control (traditional methodology) and experimental (flipped learning) groups at each educational stage (primary and secondary education). The results showed that the experimental group scored higher than the control group in academic indicators, motivation, autonomy, and interactions between different agents.

Abad, Collado-Mateo, Fernández-Espínola, Castillo, and Fuentes-Guerra [5] conducted a systematic review with a meta-analysis of the effects of technical and tactical intervention approaches on skill execution and decision-making, and they examined the influence of the teacher/coach management style. This study was performed following PRISMA guidelines (Preferred Reporting Items for Systematic Reviews and Meta-Analyses) using the Web of Science (WOS), PubMed (Medline), Scopus, and SportDiscus electronic databases. The meta-analysis results showed that tactical interventions achieved significant decision-making improvements, but they did not significantly improve skill execution compared with technical approaches. Tactical approaches are recommended for teaching games and sports in order to develop technique, understanding, tactical knowledge, and decision-making, all of which are required in gameplay.

García-Angulo, Palao, Giménez-Egido, García-Angulo, and Ortega-Toro [6] performed a quasi-experimental study on under-12 male football players to analyze the effect of reducing the number of players, the size of the goal, and the size of the playing space on the technical and tactical actions of young football players. The authors concluded that using modified rules generated a greater number of and more variability in technical–tactical actions, a greater number of actions with teammates in the pass line, greater continuity throughout the game, and more attacking and defensive actions close to the goal. This strategy also favored team play.

Trigueros et al. [7] took the version of the Scale of Basic Psychological Needs tailored to the physical exercise context and adapted it to and validated it in the Spanish PE context, with the important incorporation of novelty into the scale. In total, 2372 people took part in the research, and several analyses were performed. The results were reported for both the eight-factor structure and the higher-order double model, in which the eight subscales were joined into two constructs called frustration and satisfaction. The factorial structure of both models was invariant with respect to gender and age.

Sánchez-Oliva et al. [8] analyzed the relationships between perceived need support and need satisfaction with self-determined motivation and extracurricular physical activity intentions in the PE classroom, with sex and out-of-school sport participation included as moderators. Using multi-level analysis, the authors concluded that, at the classroom level, males benefited from need-supportive classes more than females in terms of increased autonomous motivation, whereas females benefited more than males in terms of decreased amotivation. Perceived need support at the class level moderated the negative association between need satisfaction and amotivation and between amotivation and intentions. The findings suggest that a need-supportive classroom environment may play an important role in students’ motivation and behavior.

Burgueño and Medina-Casaubón [9] performed a cluster-randomized controlled trial with 148 high school students (sport education group, *n* = 74; control group, *n* = 74) to assess the influence of sports education on sportsmanship orientations. The multivariate analysis showed significant multivariate effects at the level of each sportsmanship orientation between both groups, in favor of the sports education group. The authors concluded that sports education is an effective pedagogical model that should be considered by PE teachers for optimally promoting the moral and ethical education of high school students via the development of sportsmanship orientations in the context of school PE.

Tornero-Quiñones, Sáez-Padilla, Espina, Abad, and Sierra [10] carried out a study on 139 older people (between 65 and 87 years of age) to analyze the differences in autonomy between an active group (69 people) and a sedentary group (70 people) in terms of both basic daily activities and instrumental daily activities, as well as in functional capacity, fragility, and fall risk. By means of multivariate analysis, the authors found that the active group presented better values than the sedentary group, with statistically significant differences in all variables evaluated. Moreover, in the active group, functional capacity was a positive predictor variable of autonomy in instrumental daily activities, while fragility and fall risk were significant positive predictors of autonomy in basic daily activities. The importance of leading an active life after retirement is demonstrated once again.

García-Ceberino, Gamero, Feu, and Ibáñez [11] carried out quasi-experimental research to compare the declarative and procedural knowledge acquired by two groups of fifth-year students after implementing two intervention programs in school football: The Tactical Games Approach vs. the Direct Instruction Model. The results revealed no significant intergroup differences with regard to the methodology applied.

Muñoz-Villena, Gómez-López, and González-Hernández [12] analyzed psychological variables in 229 young male athletes from professional youth sport teams to evaluate the differences in anger expression and management according to self-esteem and perfectionism indicators. The results showed that high personal standards predicted lower anger trait indicators for athletes with low self-esteem. The results also revealed that high self-esteem acted as a protective factor in the predictive relationship between anger traits and personal standards. The study described the relationship between these variables and the young male footballers’ sense of belonging (under a high level of sports pressure). Their results highlight the need to foster athletes’ self-esteem in sports environments through prevention programs that include psychological and social resource training systems.

Thomas et al. [13] performed a randomized controlled trial in several European countries to assess whether an enriched sports activity program could increase physical fitness in a population of schoolchildren. The intervention group performed an additional warm-up protocol, which included cognitive-enhancing elements over 14 weeks, while the control group continued with the standard exercise activity. In the experimental group, the intragroup analysis (pre and post-test) showed a significant increase in the 1 kg and 3 kg ball throw, the standing broad jump, the 30 m sprint, and the Illinois agility test, while no significant differences were found in the quadruped test or the Léger shuttle run. In the control group, intragroup analysis (pre and post-test) showed no differences for any test except for the quadruped test and the Léger shuttle run.

Rodríguez-Medellín et al. [14] adapted and validated the Engagement and Disaffection Scale to the PE context in Mexico and assessed its reliability, factorial structure, and factorial invariance by gender on a sample of 1470 elementary school students. Confirmatory factor analysis, factorial invariance, internal consistency, correlations, and convergent and discriminant validity were performed. The authors concluded that the Mexican version of this scale is valid and useful for measuring these constructs in the PE context.

Noguera, Carmona, Rueda, Fernández, and Cimadevilla [15] carried out a quasi-experimental study with a recreational sample (48 healthy adults organized into two groups: 26 non-professional salsa dancers and 20 non-dancers) to evaluate whether dancing, as a physical activity that includes a lot of jumping and turning, affects spatial memory and executive functions. To do this, they used sensitive virtual reality-based tasks and the ANT-I task (Attentional Network Test-Interactions) to assess spatial memory and executive functions, respectively. Dancing integrates physical activity with music and involves the memory retrieval of complex step sequences and movements to create choreographies. The conclusion suggests that dancing can be a valid approach to slowing natural age-related cognitive decline. However, since dancing combines several factors, such as social contact, aerobic exercise, cognitive work with rhythms, and music, it is difficult to determine the weight of each of the variables analyzed.

Amado, León-del-Barco, Mendo-Lázaro, and Iglesias [16] performed a cross-sectional study with 944 school children to examine how body image satisfaction and gender can act as modulating variables on emotional intelligence in childhood. They analyzed differences in the intrapersonal, interpersonal, stress management, adaptability, and mood dimensions of emotional intelligence according to the degree of body image satisfaction and the children’s genders. The results revealed that children who were satisfied with their body image exhibited higher interpersonal intelligence, greater adaptability, and better mood; in addition, girls outperformed boys in stress management. The authors emphasized the need to promote campaigns designed by specialists to prevent body image dissatisfaction and to ensure that the benefits are able to reach the entire educational community (students, teachers, and parents). In this paper, several possibilities are described for meeting the demands of contemporary society.

Yang, Chuang, Lo, and Lee [17] propose a novel two-stage multi-criteria decision-making (MCDM) model that incorporates the concept of sustainable development into sports tourism. For this purpose, the authors carried out the Bayesian best–worst method (Bayesian BWM) to screen for important criteria and used a laboratory evaluation technique to map out complex influential relationships. To demonstrate the model’s effectiveness, it was tested in central Taiwan. The results showed that the quality of urban security, government marketing, business sponsorship, and mass transit planning were the most important criteria. Together with local festivals, this was the most influential factor overall for the evaluation system.

Pérez-Pueyo, Hortigüela-Alcalá, Hernando-Garijo, and Granero-Gallegos [18] carried out a qualitative study to propose the attitudinal style as a pedagogical model in PE. First, they defined the characteristics and elements that make up the attitudinal style as a pedagogical model; second, the authors analyzed the perceptions of future teachers regarding the usefulness and transferability of the model in their classes. The results revealed that future PE teachers considered this model to be a transcendental methodological tool for understanding and addressing PE at school. Interpersonal relationships in the classroom, student autonomy, and group responsibility were highlighted as necessary aspects with high transferability to the school.

Oliva-Lozano, Martín-Fuentes, and Muyor [19] analyzed the validity and reliability of an inertial device for monitoring the range of pelvic motion during simulated intercourse and then compared the results with those of a gold standard system. Twenty-six adults took part and were monitored during simulated intercourse using an inertial device (WIMU) and a motion capture system (gold standard). The authors concluded that WIMU could be considered a valid and reliable device for monitoring the in–out cycle range of motion during sexual intercourse in the missionary and cowgirl positions.

Conejero, Prado, Fernández-Echeverría, Collado-Mateo, and Moreno [20] performed a systematic review with meta-analysis to evaluate the scientific literature on the effect of decision training interventions/programs from a cognitive perspective on the decision-making capabilities of volleyball players. This research was carried out following PRISMA guidelines, and studies were accessed through the WOS, Pubmed (Medline), Scopus, SportDiscus, and Google Scholar databases. From the results, the authors recommend using decisional interventions or training, both as part of normal active training and as a complement to it, in order to improve players’ decision-making capabilities.

Płoszaj, Firek, and Czechowski [21] emphasized the role of referees as educators and suggested that they be taken into account when researching the educational value of sports among the youngest participants. This study was conducted on a group of 25 handball referees to analyze the quality of their interactions (a positive climate, responsiveness, behavior management, proficiency, instructing, communicating) with young players during handball matches. The authors concluded that the referees should be trained to foster a positive climate on the sports field by creating emotional ties with players (physical proximity, social conversation) while expressing an enthusiastic attitude and the joy of contact.

Kokkonen, Gråstén, Quay, and Kokkonen [22] performed structural equational modeling based on the self-reports of 363 fourth to sixth graders to analyze how students’ perceptions of their psychological environment (i.e., the motivational climate in PE) contributed to their adoption of moderate to vigorous physical activity (MVPA) via their social competence and physical activity motivation. The results showed that both the motivational climate and co-operational aspect of social competence played significant roles in students’ physical activity motivation, physical activity intention, and MVPA. Thus, the analysis of creative PE highlights that teaching behaviors contribute to students’ MVPA through motivational climates, co-operation, physical activity motivation and physical activity intention.

Carrasco-Poyatos, González-Quílez, Martínez-González-Moro and Granero-Gallegos [23] proposed a protocol study for a cluster-randomized controlled trial to assess changes in the performance of high-level athletes after a heart rate variability (HRV)-guided training period or a traditional training period and to determine the differences in athletes’ performance after both training protocols (follow-up after 12 weeks for the cluster-randomized controlled protocol, control group, and HRV group). The variables measured were the maximum oxygen uptake (VO_2max_), the maximum speed (in m/s), the maximum heart rate, the respiratory exchange ratio, ventilatory thresholds (VT1 and VT2), and their derived speed, heart rate, respiratory exchange ratio, and VO_2_ in an incremental treadmill test. To date, no other HRV-guided training research has been conducted on these types of professional athletes. It is expected that this HRV-guided training protocol will improve functional performance in high-level athletes, achieve better results than a traditional training method, and thus provide an effective strategy for coaches of high-level athletes.

Cattuzzo et al. [24] carried out a systematic review to examine studies that have assessed the performance of the supine-to-stand (STS) task in young people, adults, and the elderly. The databases accessed in the search were MEDLINE/Pubmed, Scielo, EMBASE, Scopus, ERIC/ProQuest, WOS, Science Direct, EBSCO, and Cochrane. After a qualitative analysis of the 37 studies included, the paper concluded that the STS task appears to be a universal tool for tracking functional motor competence and musculoskeletal fitness throughout life for clinical or research purposes.

Hutmacher, Eckelt, Bund, and Steffgen [25] performed a longitudinal study on 1681 students from elementary and high school in the context of PE. The measured variables were perceived need for support in PE, motivational regulation during PE, leisure time, attitude, subjective norm, perceived behavioral control, intention, and physical activity behavior. The main findings, based on mixed-effect models, revealed that the autonomy, competence, and relatedness support given by the PE teacher was positively related to autonomous motivation. In addition, longitudinal mediation analyses further supported the impact of autonomous motivation on physical activity, mediated by intention, attitude, and perceived behavioral control.

Ávalos-Ramos and Martínez-Ruiz [26] designed a qualitative study with 38 students who were in the first year of a bachelor’s degree in Physical Activity and Sport Sciences of a Spanish university and were enrolled in the Gymnastic and Artistic Skills course. The methodological design consisted of 13 practical learning sessions on the subject mentioned, in which a support strategy for autonomy in collaboration was implemented. The learning process was carried out in three phases (initial, progress, and final). The evolution of motivation, autonomy, collaboration, and achievements was highly valued throughout the process. The final assessment caused pressure and anxiety in the students, thus decreasing self-control, impairing action, and distorting the motivation experienced during the learning process.

Granero-Gallegos, González-Quílez, Plews, and Carrasco-Poyatos [27] performed a systematic review with meta-analysis to analyze the effect of HRV-guided training on VO_2max_ in endurance athletes. The methods were reported in accordance with the Campbell Collaboration policies and guidelines for systematic reviews. The register contained studies identified from the Cochrane Central Register of Controlled Trials (CENTRAL), MEDLINE, EMBASE, CINAHL Complete, the Web of Science Core Collection, Global Health, Current Contents Connect, and the SciELO Citation Index. The results showed that HRV-guided training and control training enhanced the athletes’ VO_2max_ (*p* < 0.0001), but the effect size (ES) for the HRV-guided training group was significantly higher. The amateur level and female subgroup produced better and significant results (*p* < 0.0001) for VO_2max_. HRV-guided training had a small (ES = 0.402) but positive effect on endurance athlete performance (VO_2max_), conditioned by the athlete’s level and sex.

Finally, Baños, Fuentesal, Conte, Ortiz-Camacho, and Zamarripa [28] carried out a probabilistic study on secondary school students in Mexico to analyze the mediating effect of satisfaction/enjoyment and boredom between the perception of autonomy support and academic performance in PE. The mediating effect was examined using the PROCESS V.3.5 macro. The main findings revealed that autonomy support was not a direct indicator of PE performance; instead, a forecast of positive PE performance only occurred if students felt satisfied with PE. Satisfaction with PE had a mediating effect between autonomy support and PE performance. However, boredom did not have a mediating effect between autonomy support and the student’s performance in the PE class.

## 3. Conclusions

In summary, these papers add to our understanding of the latest advances and developments in PE and sport. Through the 14 articles that analyze the educational context at different stages, from elementary school to university, the concerns of the different agents that intervene in the teaching–learning process are assessed. Two articles also focus on motivational aspects, executive functions, and spatial memory performance in relation to the practice of physical activity during leisure time. An analysis of physical activity in elderly people is also presented to address concerns such as functional capacity, frailty, and fall risk. Two articles focus on physical activity in nature and sports tourism, while another study validates the most advanced technological applications in sport and the analysis of human movements, such as the WIMU pro-device. Four papers analyze different aspects in the field of sport: football, athletes in professional youth teams, handball referees, and professional endurance athletes (runners). Finally, four systematic reviews (two with meta-analyses) explore different questions related to sports education, volleyball players, healthy individuals, and endurance athletes.
